# TLR2-Dependent Inhibition of Macrophage Responses to IFN-γ Is Mediated by Distinct, Gene-Specific Mechanisms

**DOI:** 10.1371/journal.pone.0006329

**Published:** 2009-07-24

**Authors:** Sarah A. Benson, Joel D. Ernst

**Affiliations:** 1 Department of Medicine, Division of Infectious Diseases, New York University School of Medicine, New York, New York, United States of America; 2 Department of Microbiology, New York University School of Medicine, New York, New York, United States of America; 3 Department of Pathology, New York University School of Medicine, New York, New York, United States of America; Massachusetts General Hospital/Harvard University, United States of America

## Abstract

*Mycobacterium tuberculosis* uses multiple mechanisms to avoid elimination by the immune system. We have previously shown that *M. tuberculosis* can inhibit selected macrophage responses to IFN-γ through TLR2-dependent and -independent mechanisms. To specifically address the role of TLR2 signaling in mediating this inhibition, we stimulated macrophages with the specific TLR2/1 ligand Pam_3_CSK_4_ and assayed responses to IFN-γ. Pam_3_CSK_4_ stimulation prior to IFN-γ inhibited transcription of the unrelated IFN-γ-inducible genes, CIITA and CXCL11. Surface expression of MHC class II and secretion of CXCL11 were greatly reduced as well, indicating that the reduction in transcripts had downstream effects. Inhibition of both genes required new protein synthesis. Using chromatin immunoprecipitation, we found that TLR2 stimulation inhibited IFN-γ-induced RNA polymerase II binding to the CIITA and CXCL11 promoters. Furthermore, TATA binding protein was unable to bind the TATA box of the CXCL11 promoter, suggesting that assembly of transcriptional machinery was disrupted. However, TLR2 stimulation affected chromatin modifications differently at each of the inhibited promoters. Histone H3 and H4 acetylation was reduced at the CIITA promoter but unaffected at the CXCL11 promoter. In addition, NF-κB signaling was required for inhibition of CXCL11 transcription, but not for inhibition of CIITA. Taken together, these results indicate that TLR2-dependent inhibition of IFN-γ-induced gene expression is mediated by distinct, gene-specific mechanisms that disrupt binding of the transcriptional machinery to the promoters.

## Introduction

Macrophages are important mediator cells during the immune response to invading pathogens. They are able to recognize a variety of pathogens through cell surface receptors, including members of the Toll-like receptor (TLR) family [1]. Among these receptors, TLR2 and TLR4 specifically recognize bacteria-derived lipopeptides and LPS, respectively. Engagement of TLRs results in activation of MAPK and NF-κB signaling pathways, culminating in the expression of proinflammatory cytokines and antimicrobial effector molecules [2], [3], as well as in the induction of apoptosis [4].

Macrophages also function as effector cells in the adaptive immune response. While macrophages play an important part in controlling infections as part of the innate immune response, full activation of their antimicrobial capacity and antigen presentation function only occurs after stimulation with the Th1 cytokine IFN-γ [5]. IFN-γ is essential for the control of *Mycobacterium tuberculosis* [6]–[9] and the clearance of other intracellular pathogens [10]–[13].

IFN-γ acts by binding to the heterodimeric IFN-γ receptor. Receptor binding and dimerization leads to the recruitment of JAKs 1 and 2 and ultimately to tyrosine and serine phosphorylation of the transcription factor STAT1 [14]. Phospho-STAT1 dimers then drive gene expression by binding gamma-activated sites (GAS) in the promoters of a large number of genes.

While exposure to a TLR agonist and IFN-γ can have synergistic effects and enhance activation of some IFN-γ-induced genes [15], a number of studies have shown that LPS [16], [17], whole mycobacteria [18]–[22], the mycobacterial lipoglycan phosphatidylinositol mannan [23], and mycobacterial lipoproteins [24], [25] can have inhibitory effects on a subset of IFN-γ-induced genes. This appears to be of special relevance in the context of infections with *M. tuberculosis* where, even in the presence of a strong adaptive immune response, clearance of bacteria from the infected tissue is not achieved [6], [26], [27].

Although inhibition of IFN-γ-induced gene expression by *M. tuberculosis* occurs by both TLR2-dependent and -independent mechanisms in vitro [24] and in vivo [28], we focused on the contribution of TLR2 signaling to inhibition in the experiments reported here. Pam_3_CSK_4_, a synthetic triacylated hexapeptide and specific TLR2/1 ligand [29], has been found to mimic the inhibitory effects of mycobacterial lipoproteins in macrophages [23]. Inhibition of class II transactivator (CIITA), a gene required for antigen presentation via MHC class II to CD4^+^ T cells [30], has been well characterized [18], [20], [24], [25], [31]. We wanted to extend these findings and compare inhibition of CIITA with that of CXCL11, another IFN-γ-inducible gene that we found to be strongly inhibited by Pam_3_CSK_4_ through microarray analysis. CXCL11 is a member of the CXC chemokine family and ligand for CXCR3, which is expressed on activated CD4^+^ T cells [32]. CXCL11 acts as a chemoattractant to recruit these cells to the site of inflammation [33]. Although studied during chronic *M. tuberculosis* infection [34], the role of CXCL11 during early infection is unknown.

We found that TLR2 inhibition of IFN-γ-induced transcription of CXCL11 and CIITA required new protein synthesis, but that inhibition of each of these genes involved distinct downstream mechanisms.

## Materials and Methods

### Reagents and antibodies

(S)-[2,3,-Bis(palmitoyloxy)-(2-*RS*)-propyl]-*N*-palmitoyl-(*R*)-Cys-(*S*)-Ser-(*S*)-Lys_4_-OH, 3HCl (Pam_3_CSK_4_; Calbiochem) was stored at 1 mg/ml in endotoxin-tested water (Invitrogen Life Technologies). Cycloheximide (Calbiochem) was stored at 100 mg/ml (355 mM) in DMSO. Recombinant murine IFN-γ was purchased from BD Biosciences. Polyclonal anti-acetyl-histone H3 and H4 antibodies were from Millipore. Antibodies for RNA polymerase II (N-20) and TFIID (TBP) (N-12) were purchased from Santa Cruz Biotechnology.

### Mice

C57BL/6 mice were purchased from The Jackson Laboratory, BALB/c mice were purchased from Taconic, and TNFκB (TNF^−/−^/RelA^+/−^) mice were purchased from Riken. All were maintained under specific pathogen-free conditions. All work with animals was approved by the New York University School of Medicine Institutional Animal Care and Use Committee.

### Isolation and culture of bone marrow-derived macrophages

Bone marrow-derived macrophages (BMDM) were isolated and cultured as previously described [23]. All experiments were done with C57BL/6 BMDM unless otherwise specified.

### RNA harvest and quantitative real-time PCR (qPCR)

BMDM from BALB/c mice (2×10^6^) were incubated with Pam_3_CSK_4_ (10 ng/ml) for 8 h followed by IFN-γ (20 ng/ml) for 4, 8, and 12 h. For cycloheximide experiments, BMDM from C57BL/6 mice were pretreated with DMSO or cycloheximide (500 nM) for 1 h, followed by Pam_3_CSK_4_ (10 ng/ml) for 8 h followed by IFN-γ (20 ng/ml) for 4 h in the continued absence or presence of the inhibitor. For examining NF-κB, BMDM from TNF^−/−^/RelA^+/+^ and TNF^−/−^/RelA^−/−^ mice were treated with Pam_3_CSK_4_ (10 ng/ml) for 8 h followed by IFN-γ (20 ng/ml) for 4 h. Total RNA was harvested using Qiagen RNeasy columns according to the manufacturer's directions. Genomic DNA contamination was removed by DNase treatment (Ambion). Total RNA yield was determined by nanodrop quantitation and 1 µg was reverse transcribed using the Reverse Transcription System (Promega). The cDNA equivalent of 10 ng (for GAPDH) or 50 ng (for CXCL11, CIITA, and NOS2) of total RNA was analyzed by quantitative PCR using FastStart Universal SYBR Green Master Mix (Roche) on an MJ Research Opticon 2. For quantitation, the relative values were determined by comparing the threshold cycle of each sample to a standard curve consisting of serial dilutions of a positive control cDNA sample and normalized to GAPDH. The following primers were used: CXCL11 sense, 5′-GCA CCT CTT TCA GTC TGT TTC CTG-3′; CXCL11 antisense, 5′-AGC CAT CCC TAC CAT TCA TTC AC-3′; CIITA pIV sense, 5′-GAA GTT CAC CAT TGA GCC ATT TAA-3′; CIITA pIV antisense, 5′-CTG GGT CTG CAC GAG ACG AT-3′; NOS2 sense, 5′-GTT CTC AGC CCA ACA ATA CAA GA-3′; NOS2 antisense, 5′-GTG GAC GGG TCG ATG TCA C-3′; GAPDH sense, 5′-TGT GTC CGT CGT GGA TCT GA-3′; GAPDH antisense, 5′-CCT GCT TCA CCA CCT TCT TGA-3′.

### Flow cytometry

Macrophages (2×10^6^) were plated on non-tissue culture treated plates and treated with Pam_3_CSK_4_ (10 ng/ml) for 12–15 h, followed by IFN-γ (20 ng/ml) for 24 h. Cells were harvested from plates by incubation in PBS containing 5 mM EDTA for 20 min at 4°C, then vigorous pipetting. Cells were stained with Alexa 647-conjugated anti-mouse I-A/I-E (Biolegend), washed, and resuspended in ice-cold FACS buffer (PBS, 0.1% sodium azide, 1% FCS, 500 µM EDTA). Cells were analyzed for MHC class II surface expression using a FACSCalibur (30,000 total events gated by forward and side scatter; BD Biosciences).

### ELISA

Since the CXCL11 gene in C57BL/6 mice contains a point mutation that results in the lack of CXCL11 secretion [35], BMDM from BALB/c mice were treated with Pam_3_CSK_4_ (10 ng/ml) for 8 h, followed by IFN-γ (20 ng/ml) for 4, 8, and 12 h. Culture supernatants were harvested and assayed for murine CXCL11 by ELISA according to the manufacturer's directions (R & D Systems). Samples were used neat or diluted 1∶10 or 1∶50 to allow detection within the range of the assay. Results were quantitated using an EL_x_800_UV_ spectrophotometer (Bio-Tek Instruments).

### Chromatin immunoprecipitation (ChIP)

8−10×10^6^ macrophages were treated with Pam_3_CSK_4_ (10 ng/ml) for 8–9 h, followed by IFN-γ (20 ng/ml) for 4, 8, and 12 h (for PolII and TBP binding) or 1, 2, 4, and 8 h (for histone acetylation). Cells were crosslinked by adding 1% formaldehyde for 10 min at 37°C, followed by addition of glycine (125 mM) for 5 min at room temperature. Cells were washed twice with ice-cold PBS and scraped in PBS containing protease inhibitors (Complete Mini, Roche). Fixed cells were pelleted and snap-frozen in liquid nitrogen. Samples were processed using the ChIP assay kit from Millipore (17–295). Cell pellets were thawed, resuspended in SDS lysis buffer, and lysed on ice for 10 min. Chromatin was fragmented using a Branson Digital Sonifier 250 (6 rounds at 20% amplitude for 40 s each round (0.5 s pulse, 1 s break)). One-third of the fragmented chromatin was diluted five-fold in ChIP dilution buffer. 1% of each sample was set aside as input DNA. Chromatin was immunoprecipitated overnight with anti-RNA polymerase II (10 µg), anti-TFIID (10 µg), or anti-acetylated histone H3 (5 µg) or H4 (4 µg) antibodies; the specificity of binding was determined using controls in which the primary antibody was omitted. Chromatin-antibody complexes were captured by incubation with protein A agarose beads for 1 h at 4°C, then chromatin-antibody-bead complexes were washed for 5 min at 4°C with 1 ml of each buffer in the following order: low salt immune complex wash buffer, high salt immune complex wash buffer, LiCl immune complex wash buffer, and TE (as described in the manufacturer's protocol). Chromatin was released from the beads with elution buffer (1% SDS, 0.1 M NaHCO_3_), and crosslinking was reversed by incubating input and sample chromatin in 0.2 M NaCl for 4 h at 65°C. Sample chromatin was incubated with proteinase K for 1 h at 45°C and ethanol-precipitated, then sample and input chromatin were diluted five-fold in PB buffer and purified with QiaQuick columns according to the manufacturer's protocol (Qiagen). Purified sample and input DNA was eluted with 50 µl EB buffer. 2.5 µl of eluted DNA were assayed by qPCR using primers specific for the promoter region of the assayed gene and genomic DNA as standard. ChIP output was normalized to the amount of input DNA. The resulting values for each gene were normalized using their corresponding GAPDH values. Fold enrichment is expressed in relation to the value determined for the untreated sample value. The primers used are listed in Table 1.

**Table 1:** Primers used for ChIPs.

Primer name	Sequence (5′ to 3′)
CXCL11_HISF	TCT GCC CAG AAT CCC TAC AC
CXCL11_HISR	AGA AGC CAC TGG AAG GTG AA
GAPDH_HISF	GGT CCA AAG AGA GGG AGG AG
GAPDH_HISR	AGC TAC GTG CAC CCG TAA AG
CIITA_HISF	AGC AAA CTT GGG TTG CAT GT
CIITA_HISR	TCC TGG CAG CTA TCT CAC AA
NOS2_HISF	CAC TAT TCT GCC CAA GCT GAC TTA C
NOS2_HISR	CAA TAT TCC AAC ACG CCC AGG
CXCL11_POLF	ACT GCC TGA AGA TTG CTG GT
CXCL11_POLR	ATA TTG CAG CCA GGG CTA TG
GAPDH_POLF	CCG CAT CTT CTT GTG CAG T
GAPDH_POLR	TCC CTA GAC CCG TAC AGT GC
CIITA_POLF	GAT AGC TGC CAG GAG ACT GC
CIITA_POLR	CAA ACG GGA TCT TGG AGA CA
NOS2_POLF	CCC TTT GGG AAC AGT TAT GC
NOS2_POLR	CCA AGG TGG CTG AGA AGT TT
CXCL11_TBPF	GCT GAG TGC TTT CAC CTT CC
CXCL11_TBPR	GGC TGA ACC TGA GGA GTC TG

## Results

### TLR2 stimulation inhibits IFN-γ-induced transcription of CXCL11 and CIITA, but not NOS2

**Figure 1:**
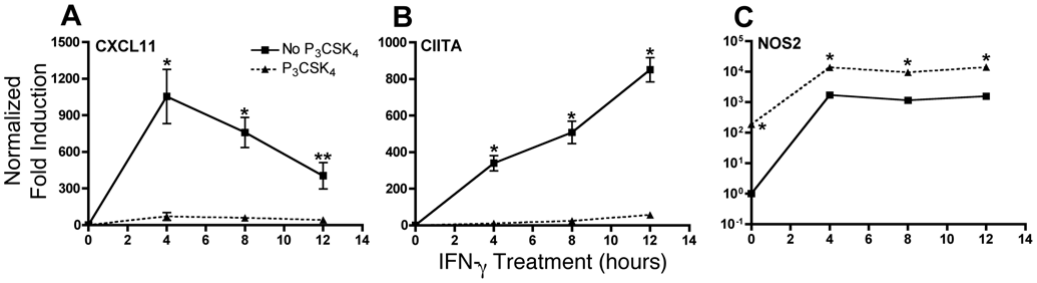
TLR2 stimulation inhibits IFN-γ-induced transcription of a subset of genes. BALB/c BMDM were treated with 10 ng/ml Pam_3_CSK_4_ for 8 h, and then 20 ng/ml IFN-γ for 4, 8, and 12 h. Total RNA was harvested, reverse transcribed, and CXCL11 (A), CIITA (B), and NOS2 (C) expression analyzed by quantitative real-time PCR (qPCR). All values were normalized to GAPDH. Results are shown as fold induction compared to untreated sample without Pam_3_CSK_4_ or IFN-γ. *, p<0.01, **, p<0.05 comparing IFN-γ alone samples with those treated with Pam_3_CSK_4_ prior to IFN-γ (as determined by two-tailed t test). Results in (A) are expressed as means±SEM of three independent experiments. Results in (B) and (C) are representative of three independent experiments. Similar results were obtained with C57BL/6 BMDM.

In the context of initial infection with *M. tuberculosis*, lung macrophages are likely to encounter the bacteria before being stimulated by IFN-γ. Therefore, we studied the effects of TLR2 stimulation by Pam_3_CSK_4_ prior to stimulation of macrophages with IFN-γ. We first analyzed the kinetics of TLR2-initiated inhibition of IFN-γ induction of CXCL11 and CIITA. Bone marrow-derived macrophages (BMDM) were treated with Pam_3_CSK_4_ for 8 h then stimulated with IFN-γ for 4, 8, and 12 h. Although CIITA and CXCL11 were both induced by IFN-γ, the kinetics of their induction differed. CXCL11 mRNA levels peaked after 4 h of IFN-γ stimulation whereas CIITA mRNA peaked after 12 h (Figs. 1A and B). Transcription of both CXCL11 and CIITA was fully inhibited in cells previously exposed to Pam_3_CSK_4_, regardless of the length of IFN-γ stimulation. As a control, we examined transcription of NOS2, an IFN-γ-inducible gene that has been found not to be inhibited by *M. tuberculosis* or mycobacterial lipoproteins [21], [24]. We found that Pam_3_CSK_4_ treatment prior to IFN-γ stimulation enhanced transcription of NOS2 in macrophages (Fig. 1C). These data show that TLR2 stimulation affects IFN-γ-induced transcription in a gene-specific manner.

### TLR2 stimulation inhibits CXCL11 protein production and MHC class II surface expression

**Figure 2:**
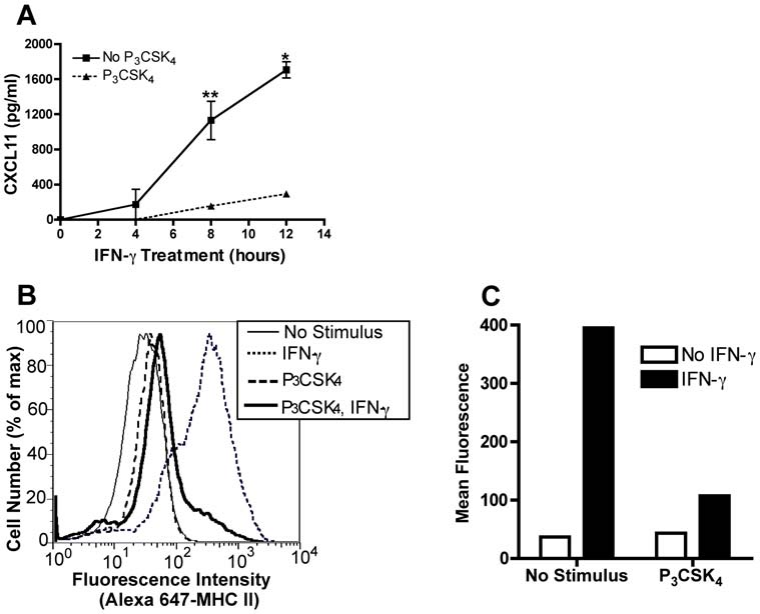
TLR2-mediated inhibition of IFN-γ induction of CXCL11 and CIITA decreases expression of protein products. A. BALB/c BMDM were treated with 10 ng/ml Pam_3_CSK_4_ for 8 h followed by 20 ng/ml IFN-γ for 4, 8, and 12 h. Culture supernatants were collected and assayed for CXCL11 protein by ELISA. *, p<0.01, **, p<0.05 comparing IFN-γ alone with Pam_3_CSK_4_ and IFN-γ treated samples (as determined by two-tailed t-test). B and C. BMDM were treated with 10 ng/ml Pam_3_CSK_4_ for 12–15 h prior to stimulation with 20 ng/ml IFN-γ for 24 h. Cells were stained with Alexa 647-conjugated anti-mouse I-A/I-E and analyzed by flow cytometry. Data shown are fluorescence intensity vs. cell number (B) and mean I-A/I-E fluorescence (C). Results are expressed as means±SEM from two independent experiments (A) and are representative of at least five independent experiments (B and C).

Since transcription of CXCL11 and CIITA was significantly reduced in TLR2 stimulated macrophages, we determined whether inhibition was reflected by reduction of the protein products of these genes. To examine the effect on CXCL11, we treated BALB/c BMDM with Pam_3_CSK_4_ for 8 h followed by IFN-γ for 4, 8, and 12 h. Culture supernatants were harvested and assayed for CXCL11 by ELISA. Stimulation with IFN-γ resulted in secretion of CXCL11 after 8 and 12 h of treatment (Fig. 2A). However, prior TLR2 stimulation inhibited IFN-γ-induced CXCL11 protein levels by over 80% at both of these time points.

To examine the downstream effect of TLR2 stimulation on CIITA, we measured surface expression of MHC class II, whose expression is regulated by, and depends on, CIITA. BMDM were treated with Pam_3_CSK_4_ for 12–15 h then stimulated with IFN-γ for 24 h (the minimal time needed for increased MHC class II surface expression). IFN-γ stimulation alone caused an eleven-fold increase of MHC class II on the cell surface (Fig. 2B). However, Pam_3_CSK_4_ treatment prior to IFN-γ inhibited this upregulation by 73%, as assessed by mean fluorescent intensity (Fig. 2C). Pam_3_CSK_4_ alone (without IFN-γ) had no effect on surface MHC class II levels. These results indicate that TLR2-mediated inhibition of CXCL11 and CIITA transcription has downstream consequences at the protein level.

### TLR2-mediated inhibition of CXCL11 and CIITA requires new protein synthesis

**Figure 3:**
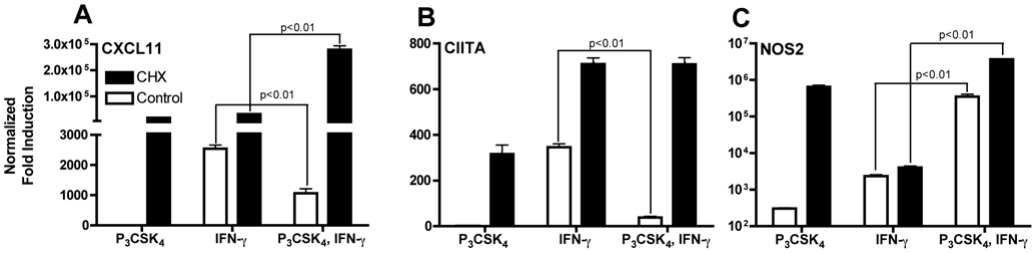
New protein synthesis is required for inhibition of CIITA and CXCL11. BMDM were pretreated with 500 nM cycloheximide or DMSO for 1 h prior to treatment with 10 ng/ml Pam_3_CSK_4_ for 8 h followed by 20 ng/ml IFN-γ for 4 h in the continued presence or absence of inhibitor. Total RNA was harvested after IFN-γ stimulation. CXCL11 (A), CIITA (B), and NOS2 (C) expression was assayed by qPCR and normalized to GAPDH and untreated samples. The concentration of cycloheximide used inhibited TNF production (as a measure of protein synthesis) by over 90% with minimal cell death. Statistical significance between IFN-γ alone samples and those treated with Pam_3_CSK_4_ prior to IFNγ was determined by two-tailed t-test.

We have previously shown that *M. tuberculosis*-mediated inhibition of CIITA induction by IFN-γ requires new protein synthesis [23]. We extended these findings and examined the effect of cycloheximide, a pharmacological inhibitor of protein synthesis, on induction of CXCL11, CIITA, and NOS2 mRNA by IFN-γ in TLR2 stimulated macrophages. In control samples, TLR2 stimulation prior to IFN-γ inhibited CXCL11 and CIITA induction, but enhanced NOS2 induction, as previously seen. However, treatment with cycloheximide fully reversed inhibition of CXCL11 and CIITA and further enhanced NOS2 expression (Fig. 3). These results indicate that TLR2 stimulation induces production of one or more proteins that are required for inhibition of macrophage gene expression in response to IFN-γ. Cycloheximide treatment also greatly enhanced expression of all three genes, with the strongest effect on CXCL11, presumably due to the lack of a negative regulatory protein.

### TLR2 stimulation prevents RNA polymerase II from binding the CXCL11 and CIITA promoters

**Figure 4:**
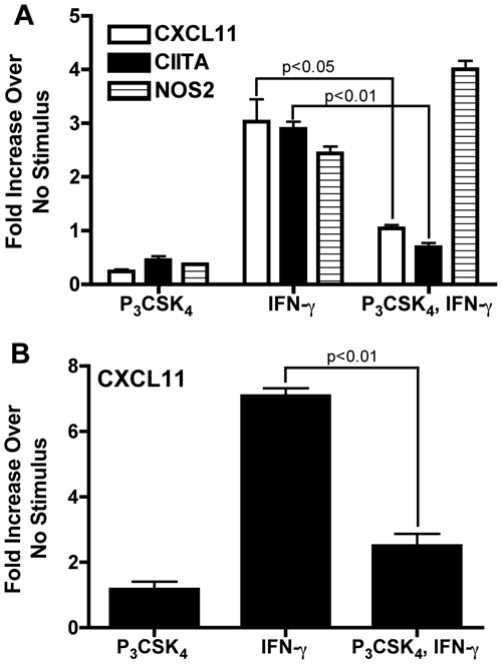
TLR2 stimulation prevents binding of general transcriptional machinery to the CIITA and CXCL11 promoters. BMDM were treated with 10 ng/ml Pam_3_CSK_4_ for 8–9 h, then 20 ng/ml IFN-γ for 4 h. Cross-linked DNA was sheared and immunoprecipitated with anti-PolII (A) or anti-TBP (B) antibodies. Precipitated and input DNA for each sample were assayed by qPCR with primers specific for the transcriptional start site in the promoters of CXCL11, CIITA, and NOS2 (A) or the TATA box of the CXCL11 promoter (B). All values were normalized to GAPDH. Results are expressed as fold increase over untreated controls and are the mean of triplicate samples±SD. Statistical significance between IFN-γ alone samples and Pam_3_CSK_4_ prior to IFN-γ treated samples was determined by two-tailed t-test. Results are representative of at least two independent experiments.

To further characterize the mechanism of inhibition of transcriptional responses to IFN-γ by prior TLR2 stimulation, we determined whether RNA polymerase II (PolII) binds the promoters of the affected genes. We treated BMDM with Pam_3_CSK_4_ for 8 h then stimulated with IFN-γ for 4 h, followed by chromatin immunoprecipitation (ChIP) to assay binding of PolII to the CXCL11, CIITA, and NOS2 promoters. Primers that specifically amplified regions flanking the transcriptional start site of each gene were designed to detect initial binding of PolII to the promoters. IFN-γ stimulation induced binding of PolII to the CXCL11 and CIITA promoters, as indicated by a three-fold increase in pulldown of promoter fragments of these genes (Fig. 4A). However, stimulation of TLR2 prior to IFN-γ inhibited binding of PolII by 66% (CXCL11) and 76% (CIITA) compared with IFN-γ alone. The effect of TLR2 stimulation was not merely to delay IFN-γ-stimulated PolII binding at these promoters, as similar inhibition of binding was seen after 8 and 12 h of IFN-γ stimulation (data not shown). As a control, we assayed PolII binding at the NOS2 promoter, since TLR2 stimulation did not decrease NOS2 mRNA induction by IFN-γ (Fig. 1C). IFN-γ stimulation alone caused a two-fold increase in binding and prior Pam_3_CSK_4_ treatment resulted in a further increase in binding (Fig. 4A). This was similar to the effect observed at the level of transcription (Fig. 1C). These results indicate that TLR2 stimulation prevents PolII from binding to the CXCL11 and CIITA promoters, but does not affect binding to the NOS2 promoter. The effect of TLR2 stimulation on transcription of IFN-γ-responsive genes correlates with PolII binding at the respective promoters.

### TLR2 stimulation prevents binding of TBP to the CXCL11 promoter

Since we found that TLR2 stimulation prevents PolII from binding the CXCL11 and CIITA promoters, we determined whether another member of the general transcriptional machinery was similarly affected. We examined the ability of TATA binding protein (TBP) to bind the TATA box in the CXCL11 promoter following Pam_3_CSK_4_ and IFN-γ stimulation. The CIITA promoter lacks a TATA box (our unpublished observation), so we did not include it in our experiments. IFN-γ stimulation resulted in a five to seven-fold increase in TBP binding to the CXCL11 promoter after 4, 8, and 12 h (Fig. 4B and data not shown). However, TLR2 stimulation prior to IFN-γ decreased TBP binding to the CXCL11 promoter by 65%. This suggests that the lack of transcription is not due to a deficiency in PolII binding alone, but that prolonged TLR2 signaling also inhibits binding of other members of the general transcriptional machinery.

### TLR2 signaling inhibits histone acetylation at the CIITA promoter, but not at the CXCL11 promoter

**Figure 5:**
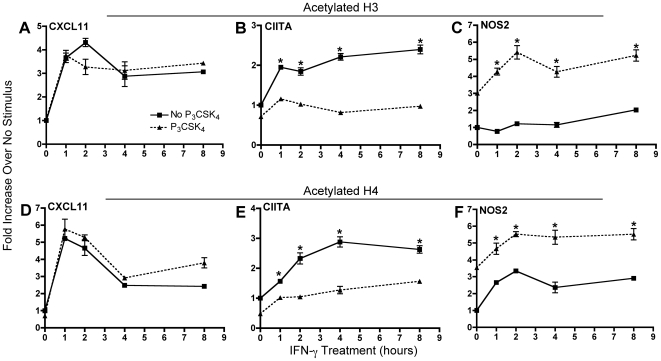
TLR2 stimulation inhibits histone acetylation at the CIITA promoter, but not the CXCL11 promoter. BMDM were treated with 10 ng/ml Pam_3_CSK_4_ for 8 h prior to stimulation with 20 ng/ml IFN-γ for 1, 2, 4, and 8 h. Cross-linked, sheared DNA was immunoprecipitated with antibodies against either acetylated histone H3 (A, B, C) or H4 (D, E, F). Precipitates were analyzed by qPCR using primers specific for the CXCL11 (A, D), CIITA (B, E), and NOS2 (C, F) promoters. Values were normalized to GAPDH and untreated controls and are the mean of triplicate samples±SD. *, p<0.01 comparing IFN-γ alone with Pam_3_CSK_4_ prior to IFN-γ treated samples (as determined by two-tailed t-test). Results are representative of two independent experiments.

Transcriptional activity is usually associated with increased acetylation of histones H3 and H4 in the promoter region of transcribed genes [36], and TLR2 stimulation by the mycobacterial lipoprotein LpqH inhibits acetylation of histones H3 and H4 at the CIITA promoter after 4 h of IFN-γ stimulation in macrophages [25]. We extended these findings by examining earlier IFN-γ time points and by determining whether TLR2 stimulation also inhibited histone acetylation at the CXCL11 promoter. BMDM were treated with Pam_3_CSK_4_ for 8 h followed by IFN-γ for 1, 2, 4, and 8 h. ChIPs were then performed using antibodies against either acetylated histone H3 or acetylated histone H4. At the CXCL11 promoter, we observed a rapid increase in acetylation of both histones after 1 h of IFN-γ stimulation, which persisted for as long as IFN-γ was present (Figs. 5A and D). TLR2 stimulation prior to IFN-γ did not inhibit histone acetylation at the CXCL11 promoter at any time point after IFN-γ stimulation. In contrast, TLR2 stimulation significantly inhibited histone H3 and H4 acetylation at the CIITA promoter by 35–63% at all IFN-γ time points (Figs. 5B and E). For comparison, we also examined histone acetylation at the NOS2 promoter. TLR2 stimulation prior to IFN-γ resulted in an increase of histone acetylation over IFN-γ stimulation alone (Figs. 5C and F). These data indicate that TLR2-mediated inhibition of IFN-γ induction of CIITA and CXCL11 occurs by at least two distinct mechanisms, one that affects histone acetylation and one that does not.

### NF-κB is required for TLR2-mediated inhibition of CXCL11, but not CIITA, transcription

**Figure 6:**
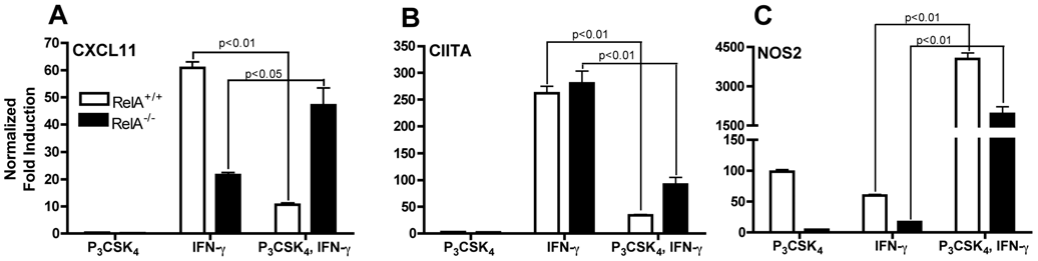
IFN-γ-induced transcription of CXCL11, but not CIITA, is restored in TLR2 stimulated RelA^−/−^ macrophages. BMDM from TNF^−/−^/RelA^+/+^ and TNF^−/−^/RelA^−/−^ mice were treated with 10 ng/ml Pam_3_CSK_4_ for 8 h followed by 20 ng/ml IFN-γ for 4 h. Total RNA was harvested, reverse transcribed, and CXCL11 (A), CIITA (B), and NOS2 (C) expression analyzed by qPCR. All values were normalized to GAPDH and shown as fold induction compared to untreated samples without Pam_3_CSK_4_ or IFN-γ. Statistical significance was determined by two-tailed t-test between IFN-γ alone with Pam_3_CSK_4_ prior to IFN-γ samples. C57BL/6 BMDM showed similar results to those obtained with TNF^−/−^/RelA^+/+^ BMDM.

Transcriptional responses to TLR2 activation are largely mediated by the transcription factor NF-κB, a heterodimer commonly consisting of the RelA and p50 subunits. RelA deficiency is embryonic lethal but can be rescued by deletion of TNF [37], [38]. We examined the role of NF-κB in TLR2-mediated inhibition of responses to IFN-γ using macrophages from TNF^−/−^/RelA^−/−^ and TNF^−/−^/RelA^+/+^ mice. BMDM were treated with Pam_3_CSK_4_ for 8 h followed by IFN-γ for 4 h. TLR2 stimulation prior to IFN-γ inhibited induction of CXCL11 and CIITA in RelA^+/+^ macrophages (Figs. 6A and B). In RelA^−/−^ macrophages, CXCL11 induction by IFN-γ was fully restored despite prior TLR2 stimulation (Fig. 6A). However, CIITA induction was still significantly reduced in these cells (Fig. 6B). TNF deficiency did not affect transcriptional responses of CXCL11 or CIITA, as results with TNF^−/−^/RelA^+/+^ macrophages were similar to C57BL/6 macrophages (data not shown). However, lack of TNF did result in lower NOS2 expression that was further decreased in RelA^−/−^ macrophages (Fig. 6C). These data provide further support that CXCL11 and CIITA are differentially regulated upon TLR2 stimulation. Inhibition of CXCL11 induction requires NF-κB whereas inhibition of CIITA does not.

## Discussion

*M. tuberculosis* survives in macrophages, even when they are stimulated with IFN-γ [39], [40]. We and others have found that *M. tuberculosis* blocks selected macrophage responses to IFN-γ by inhibiting transcription of a subset of IFN-γ-inducible genes [18], [20]–[22]. At least two proximal mechanisms are involved. One is initiated by mycobacterial peptidoglycan in a TLR2- and MyD88-independent manner [24], while the other requires TLR2 and MyD88 and is initiated by lipoproteins and phosphatidylinositol mannan [23], [24]. In the experiments presented here, we examined the mechanisms downstream of TLR2 in mediating this inhibition, by using the specific TLR2/1 agonist Pam_3_CSK_4_.

This work expands on previous studies that examined inhibition of CIITA transcription [18], [20], [24], [25], [31], by comparing the kinetics of inhibition of CIITA with that of CXCL11, an unrelated IFN-γ-inducible gene, as well as with NOS2, an IFN-γ-inducible gene that is not inhibited by *M. tuberculosis* [21], [24]. We found that TLR2 stimulation inhibited IFN-γ-induced transcription of CIITA and CXCL11 to similar levels over a time course of IFN-γ stimulation (Figs. 1A and B), indicating that macrophages are unable to recover the ability to respond to IFN-γ, regardless of the length of stimulation. This reduction in transcription resulted in decreased CXCL11 secretion and decreased MHC class II on the macrophage surface (Fig. 2). However, TLR2 stimulation resulted in an increase in NOS2 mRNA (Fig. 1C), indicating that TLR2-mediated transcriptional inhibition is gene specific, and therefore not mediated by inhibition of a proximal signaling step such as STAT1 activation.

One of the initial steps in transcription initiation is acetylation of lysine residues within the N-terminal tails of core histones. This decreases their affinity for DNA, allowing a more permissive chromatin structure for transcription factors and other proteins to bind to DNA [41]. This process is tightly controlled by two counteracting enzymatic activities: the histone deacetylases (HDACs) and the histone acetyltransferases (HATs). HDACs repress transcription by removing acetyl groups whereas HATs acetylate critical lysines.

Mammalian HDACs fall into three classes (I, II, and III) based on sequence homology to yeast HDACs [42]. Class II and III HDACs are expressed in a limited number of tissues. However, HDACs 1 and 2 (of class I) appear to be constitutively expressed [43]–[47]. Therefore, their activity must be tightly regulated. They are the enzymatically active components of multi-protein complexes, which include DNA binding proteins and corepressors. These complexes target the HDACs to the promoters of genes by interactions with sequence-specific transcription factors, leading to transcriptional repression of select genes. HDACs require complex formation for enzymatic activity, as most purified recombinant HDACs are inactive [48], [49]. In addition to regulation by protein-protein interactions, HDACs can be post-translationally modified. Casein kinase 2 (CK2), a ubiquitously expressed protein kinase, has been identified as a key regulator of class I HDACs [50], [51]. CK2 activity is induced by a number of stimuli including IFN-γ and the TLR4 ligand LPS [52]. CK2 phosphorylation of S421/S422 and S423/S424 at the C-terminal region of HDACs 1 and 2, respectively, is important for complex formation and enzymatic activity. Although more light is being shed on how HDAC activity is regulated, many of the signaling pathways involved remain to be elucidated.

HATs are a diverse group of enzymes that regulate transcription by rendering the chromatin more accessible via acetylation of histone tails. Gene transcription in response to IFN-γ involves CREB binding protein (CBP) and/or p300, coactivators that have HAT activity [53]. Following IFN-γ stimulation, phosphorylated STAT1 associates with CBP/p300, which is thought to facilitate contact with transcriptional machinery at the promoter regions of IFN-γ-inducible genes [54]. Similar to HDACs, the HAT activity of CBP and p300 is tightly regulated via interactions with other proteins as well as by phosphorylation by a number of kinases. Phosphorylation by p42/p44 MAPK, CDK2, protein kinase A, and IKKα upregulate HAT activity [53], [55] whereas phosphorylation by protein kinase Cδ reduces HAT activity [56].

Transcriptional repressors also regulate gene expression. General transcriptional repression occurs when a repressor sequesters or modifies a member of the general transcriptional machinery or PolII itself [57]. Expression of all genes transcribed by PolII will then be inhibited. Gene-specific repression occurs when a repressor targets a specific coactivator or interacts in a promoter-specific manner with members of the general transcriptional machinery or PolII. Therefore, only a subset of genes will be inhibited.

Previously published data suggest that gene-specific repressors may contribute to TLR2-initiated inhibition of transcriptional responses to IFN-γ [31]. The mycobacterial lipoprotein and TLR2 agonist LpqH induces expression of the transcription factor C/EBPβ, which can act as a transcriptional activator or repressor, depending on the promoter and stimulus. This increased expression correlated with inhibition of IFN-γ-induced CIITA transcription, and macrophages stimulated with LpqH and IFN-γ exhibited increased C/EBPβ binding to the CIITA promoter. The NOS2 promoter also has a C/EBPβ binding site that is involved in gene induction in response to TLR and IFN-γ stimulation [58]. The CXCL11 promoter has two potential C/EBPβ binding sites from −52 to −44 (TGCCTGAAG) and from −24 to −16 (TCCTCAGAC), although the functionality of these sites remains to be determined. It is therefore possible that C/EBPβ or a related protein may contribute to negative regulation of IFN-γ-induction of both CIITA and CXCL11. However, C/EBPβ^−/−^ macrophages were found to remain sensitive to LpqH-mediated transcriptional inhibition of CIITA [31], suggesting that additional factors are involved. One such factor may be C/EBPδ, whose expression is induced by TLR4 stimulation and has been shown to regulate genes involved in the innate immune response as part of a circuit with other transcription factors [59]. TLR2 stimulation also induces C/EBPδ expression and binding to the CIITA promoter [31]. However, the potential inhibitory function of C/EBPδ on IFN-γ-induced transcription needs to be explored.

We attempted to identify other potential transcription factor binding sites that might be responsible for TLR2-mediated inhibition by comparing the promoter sequences of several IFN-γ-inducible, Pam_3_CSK_4_-inhibited genes. Computer-based comparison of these promoter sequences with those from a control group of unaffected, IFN-γ-inducible genes did not yield an over-represented transcription factor binding motif, indicating that more than one signaling pathway is involved, or that inhibition is mediated by one or proteins that do not bind promoter elements directly (data not shown).

However, when specifically examining the CXCL11 and CIITA promoters, we found that CXCL11 has an NF-κB binding site at −68 to −59 (GGGGAATTCC) that is missing in CIITA. Further investigation of the role of NF-κB in TLR2-mediated inhibition of these genes using RelA^−/−^ macrophages showed that inhibition of CXCL11, but not CIITA, is NF-κB dependent (Fig. 6). We could not detect binding of p65 or p50 to this site in the CXCL11 promoter (data not shown), suggesting that NF-κB most likely has an indirect inhibitory effect, possibly by inducing expression of a protein that blocks CXCL11 transcription upon TLR2 stimulation. The reversal of transcriptional inhibition seen when new protein synthesis is blocked is concordant with this mechanism (Fig. 3A).

We also examined potential epigenetic mechanisms as TLR2 stimulation has been shown to inhibit IFN-γ-induced histone acetylation at the promoters of genes involved in MHC class II antigen presentation [25], [43]. ChIP experiments done in murine macrophages stimulated with LpqH followed by IFN-γ showed that acetylation of histones H3 and H4 was reduced at the CIITA promoter compared to IFN-γ stimulation alone [25]. This inhibition was abrogated with pharmacological blockade of MAPKs. In addition, this inhibition was partially reversed in the presence of the HDAC inhibitor sodium butyrate, suggesting that inhibition of histone acetylation is one mechanism by which TLR2 stimulation prevents CIITA expression.

We elaborated on those experiments to determine if inhibition of histone acetylation was a common mechanism for TLR2-mediated inhibition of other IFN-γ-inducible genes. In contrast to the inhibition of IFN-γ-induced histone acetylation at the CIITA promoter (Fig. 5B and E), histone acetylation at the CXCL11 promoter was unaffected by TLR2 stimulation (Fig. 5A and D). Concordant with the transcriptional data, histone acetylation at the NOS2 promoter increased with TLR2 stimulation (Fig. 5C and F). This indicates that a decrease in modifications that make the chromatin more accessible to transcription factors and coactivators may be involved, but this is not the sole mechanism responsible for TLR2-mediated inhibition of transcriptional responses to IFN-γ.

In contrast to the gene-selective requirement for NF-κB and different effects of TLR2 stimulation on histone acetylation, we found that TLR2 stimulation decreased IFN-γ-induced binding of RNA polymerase II at both the CIITA and CXCL11 promoters, but not at the NOS2 promoter (Fig. 4A). This indicates that while the intermediate signaling steps may vary for distinct genes, TLR2 stimulation interrupts a crucial step in transcription initiation at specific IFN-γ-responsive genes.

In addition to *M. tuberculosis*, other pathogens have developed mechanisms to evade immune responses through disruption of host gene transcription. The intracellular bacteria *Listeria monocytogenes* induces a reduction in total cellular histone acetylation early after infection, mediated partially by listeriolysin O [60]. The opportunistic pathogen *Mycobacterium avium* inhibits histone acetylation at the HLA-DRα promoter, possibly through recruitment of HDAC corepressor mSin3a, which was found to bind the promoter following infection [43]. In those studies, infection also led to a reduction in CBP recruitment to the HLA-DRα promoter.

Viruses also disrupt host gene transcription by preventing transcriptional machinery assembly at gene promoters. Poliovirus cleaves general transcription factors [61], Rift Valley Fever virus blocks transcription factor assembly [62], and vesicular stomatitis virus targets TBP using an unknown mechanism [63]. These all prevent general PolII transcription. However, murine cytomegalovirus has been shown to inhibit specific gene transcription by targeting IFN-γ-inducible genes without affecting JAK/STAT activation [64]. PolII binding to the promoters of the affected genes was significantly reduced, suggesting that disruption of transcriptional machinery assembly was responsible for this inhibition.

The findings reported here extend the understanding of the mechanisms that may be used by at least one pathogen, *Mycobacterium tuberculosis*, to evade elimination by adaptive immune responses. Developing the means to increase the efficacy of adaptive immune responses in order to better control infection with *M. tuberculosis* will require additional investigation; increasing the efficacy of IFN-γ by restoring macrophage transcriptional responses to this cytokine may be one effective approach, but will require further study.
